# Supplementary material for: Splice-Junction-Based Mapping of Alternative Isoforms in the Human Proteome
Source: Cell Rep. Author manuscript; Available in PMC 2020 Jan 15. (PMC6961840; doi:10.1016/j.celrep.2019.11.026)
Supplement: 2 [file NIHMS1546469-supplement-2.zip › DF1/PXD006675/LeftAtrium/LeftAtrium_2_MYBPC3_RTDSHEDTGILDFSSLLK.pdf]

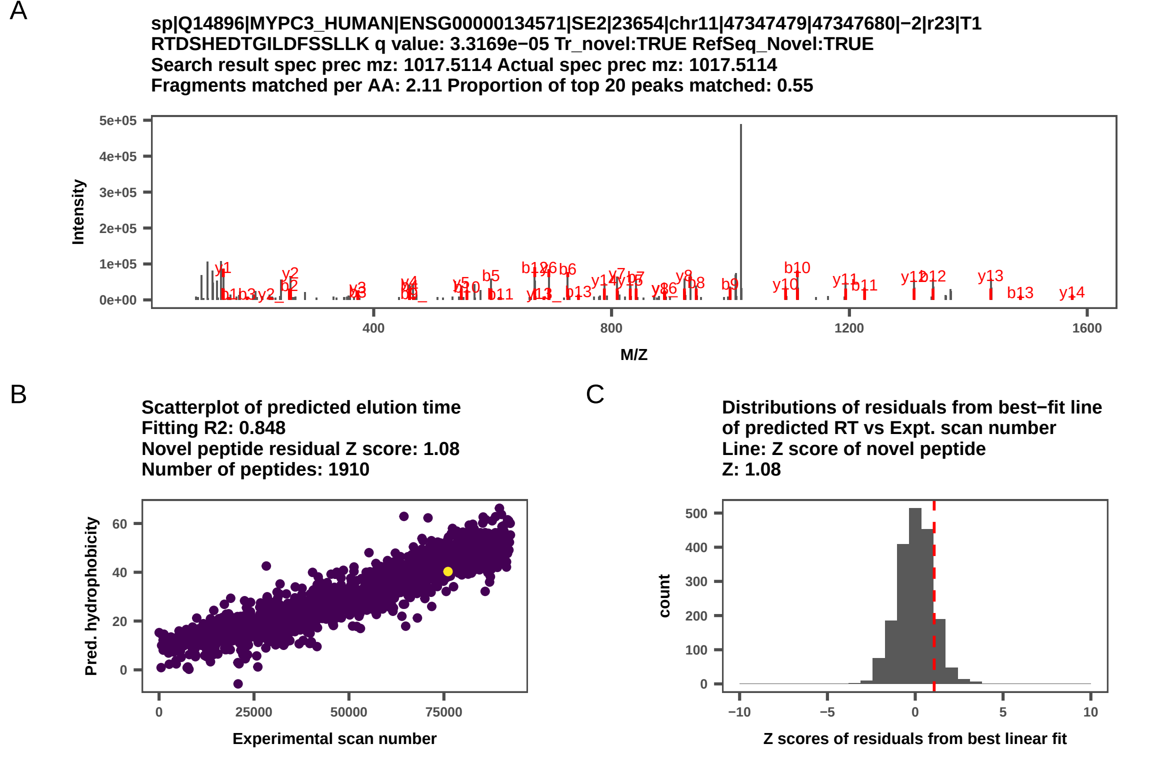
A
sp|Q14896|MYPC3_HUMAN|ENSG00000134571|SE2|23654|chr11|47347479|47347680|−2|r23|T1
RTDSHEDTGILDFSSLLK q value: 3.3169e−05 Tr_novel:TRUE RefSeq_Novel:TRUE
Search result spec prec mz: 1017.5114 Actual spec prec mz: 1017.5114
Fragments matched per AA: 2.11 Proportion of top 20 peaks matched: 0.55
Intensity
5e+05
4e+05
3e+05
2e+05
1e+05
0e+00
400
800
1200
1600
M/Z
B
Scatterplot of predicted elution time
Fitting R2: 0.848
Novel peptide residual Z score: 1.08
Number of peptides: 1910
Pred. hydrophobicity
60
40
20
0
25000
50000
75000
Experimental scan number
C
Distributions of residuals from best−fit line
of predicted RT vs Expt. scan number
Line: Z score of novel peptide
Z: 1.08
count
500
400
300
200
100
−10
−5
5
10
Z scores of residuals from best linear fit
